# Long non-coding RNA HOXA-AS2 promotes proliferation and invasion of breast cancer by acting as a miR-520c-3p sponge

**DOI:** 10.18632/oncotarget.17552

**Published:** 2017-05-02

**Authors:** Yu Fang, Jingxuan Wang, Feng Wu, Ying Song, Shu Zhao, Qingyuan Zhang

**Affiliations:** ^1^ Department of Medical Oncology, The Affiliated Tumor Hospital of Harbin Medical University, Nangang District, Harbin 151000, Heilongjiang Province, China; ^2^ Department of Gastroenterology, The First Affiliated Hospital of Harbin Medical University, Nangang District, Harbin 151000, Heilongjiang Province, China

**Keywords:** LncRNA-HOXA-AS2, miR-520c-3p, breast cancer, growth, metastasis

## Abstract

The long non-coding RNA (lncRNA) HOXA cluster antisense RNA2 (HOXA-AS2) has recently been shown to be dysregulated and involved in the progression of several cancers. However, the biological role and clinical significance of HOXA-AS2 in the carcinogenesis of breast cancer are still unclear. In the present study, we found that HOXA-AS2 was up-regulated in human breast cancer tissues and cell lines and associated with clinicopathological characteristics. Silencing of HOXA-AS2 inhibited the progression of breast cancer cells *in vitro* and *in vivo*. Furthermore, microarray profiling indicated that HOXA-AS2 serves as an endogenous sponge by directly binding to miR-520c-3p and down-regulating miR-520c-3p expression. We demonstrated that HOXA-AS2 controls the expression of miR-520c-3p target genes, TGFBR2 and RELA, in breast cancer cells. Therefore, our study may provide a better understanding of the pathogenesis of breast cancer and suggests that HOXA-AS2 may be a potential prognostic and therapeutic target in breast cancer.

## INTRODUCTION

Breast cancer is a malignant tumor originating from breast tissue and is the most common cancer in women worldwide [1, 2]. Recently, the development of early diagnoses, radical surgery and adjuvant therapy has decreased the mortality rates of breast cancer [3–5]; however, it is still the most common gynecologic cancer. In China, the growing incidence of breast cancer threatens women's health and has gradually become a social problem [6]. Research has shown that many molecular triggers play significant roles in breast cancer development and chemotherapy resistance [7]. However, the molecular regulatory network of breast cancer is very complex, and the molecular mechanisms of breast cancer progression needed to be further explored.

In the human genome, only 1-2% of genes encode proteins [8], and the rest of the genome encodes a large amount of non-coding RNAs (ncRNA) [9].

LncRNAs are a class of RNA molecules that are longer than 200 nucleotides and cannot be translated into proteins [10]. Research has shown that lncRNAs are involved in a variety of biological processes, such as development [11], apoptosis [12, 13], proliferation and differentiation [14], and carcinogenesis [15–17]. Recent studies have also found that lncRNAs are abnormally expressed in a variety of cancers, including breast cancer [18], lung cancer [19], hepatocellular carcinoma [20], and gastric cancer [21]. Therefore, lncRNAs are regarded as potential biomarkers and may lead to a new treatment era for various cancers [22, 23]. HOXA cluster antisense RNA 2 (HOXA-AS2) is an lncRNA located between the HOXA3 and HOXA4 genes in the HOXA cluster. Recent studies indicated that HOXA-AS2 represses apoptosis in NB4 promyelocytic leukemia cells treated with trans retinoic acid [24], promotes the proliferation of gastric cancer via P21/PLK3/DDIT3 [25] and accelerates tumorigenesis of hepatocellular carcinoma [26]. However, the role of HOXA-AS2 in breast cancer development, invasion and metastasis remains unknown.

Many studies have shown that lncRNAs can serve as a competing endogenous RNAs (ceRNAs) to regulate microRNAs (miRNAs) [27, 28]. CeRNAs play a post-transcriptional regulatory role in miRNA distribution on their targets. MiRNAs are a class of non-coding RNA of approximately 20 nucleotides and regulate gene and protein expression by targeting the 3′ untranslated region (UTR) of mRNAs [29, 30]. Previous studies have shown that miRNAs are involved in post-transcriptional regulation of various biological processes [31–33]. In addition, miRNAs serve as tumor suppressor genes or oncogenes and play important roles in the occurrence and development of human cancers [31, 34]. In this study, we aimed to explore the ceRNA mechanism of HOXA-AS2 though miR-520c-3p and revealed the functional relevance of miR-520c-3p and HOXA-AS2 in breast cancer.

## RESULTS

### LncRNA-HOXA-AS2 was up-regulated in human breast cancer tissues and cell lines

**Figure 1:**
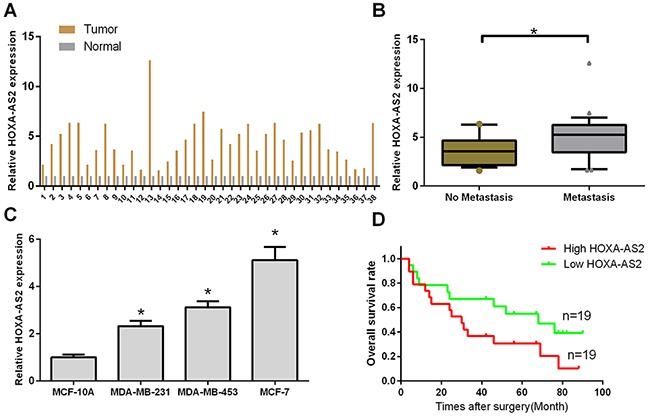
HOXA-AS2 was up-regulated in human breast cancer tissues and cell lines **(A)** The expression level of HOXA-AS2 was detected by qRT-PCR and normalized to GAPDH in human breast cancer tissues (tumor, n = 38) and adjacent normal tissues (Normal, n = 38). **(B)** Relative expression level of HOXA-AS2 was measured by qRT-PCR in breast cancer tissues with (n = 15) or without distant metastasis (n = 23). *P < 0.05. **(C)** Relative expression level of HOXA-AS2 was analyzed in a human mammary epithelial cell line (MCF-10A) and four breast cancer cell lines (MDA-MB-231, MDA-MB-453, and MCF-7) by qRT-PCR, and GAPDH was used as an internal control (*P < 0.05). **(D)** Kaplan-Meier curve of overall survival in breast cancer patients with high HOXA-AS2 levels (n = 19) and low HOXA-AS2 levels (n = 19).

The expression level of HOXA-AS2 was detected by qRT-PCR in 38 pairs of breast cancer tissues (Tumor) compared with corresponding adjacent normal tissues (Normal), and we found that HOXA-AS2 expression was significantly up-regulated in tumor tissues compared with normal tissues (Figure 1A). Next, we found that high expression of HOXA-AS2 was corrected with distant metastasis (n = 16) (*P < 0.05, Figure 1B). In addition, the relationships between HOXA-AS2 expression and clinical characteristics in 38 patients with breast cancer were analyzed, and we found that expression was significantly associated with invasion (*P* = 0.0005), lymphatic metastasis (*P* < 0.0001), distant metastasis (*P* < 0.0001), and TNM stage (*P* < 0.0001) but not related to age (Table 1). Further analysis showed that HOXA-AS2 was significantly higher in breast cancer cell (MDA-MB-231, MDA-MB-453, and MCF-7) lines than that of the non-metastatic human mammary epithelial cell line MCF-10A (*P* < 0.05, Figure 1C). Moreover, high HOXA-AS2 expression was associated with poor overall survival compared to that of patients with low HOXA-AS2 (*P* < 0.05, Figure 1D). These results suggested that HOXA-AS2 might be an oncogene in breast cancer.

**Table 1:** Correlation analysis between HOXA-AS2 expression and clinic pathological characteristics of patients with breast cancer

Characteristics	No. of patients	Mean ± SD	*P* value
**Total no. of patients**	38		
**Age (year)**			
>60	28 (73.7%)	11.15 ± 1.02	0.885
≤60	10 (26.3%)	11.21 ± 1.37	
**Invasion**			
T0-T2	21 (55.3%)	12.79 ± 1.31	0.0005***
T3-T4	17 (44.7%)	11.28 ± 1.07	
**Lymphatic metastasis**			
N0	23 (60.5%)	13.64 ± 1.21	<0.0001***
N1-N3	15 (39.5%)	10.35 ± 0.96	
**Distant metastasis**			
M0	27 (71.1%)	12.89 ± 1.13	<0.0001***
M1	11 (28.9%)	10.92 ± 1.17	
**TNM stage**			
0 & I & II	23 (60.5%)	13.64 ± 1.21	<0.0001***
III & IV	15 (39.5%)	10.35 ± 0.96	

***Indicated statistical significance (*P* < 0.001).

### Silencing of HOXA-AS2 inhibits the progression of breast cancer cells

**Figure 2:**
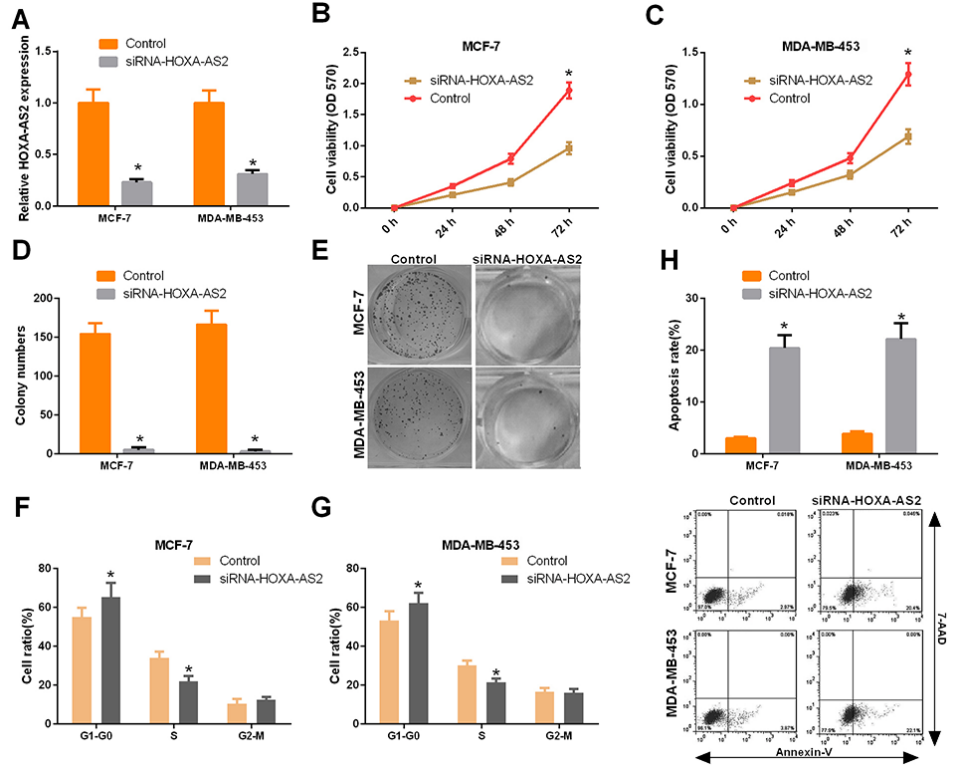
Silencing of HOXA-AS2 inhibited the progression of breast cancer **(A)** MCF-7 and MDA-MB-453 cells were transfected with HOXA-AS2 siRNAs or control for 48 hrs using Lipofectamine 3000. The relative HOXA-AS2 expression level was determined by qRT-PCR with the comparative delta-delta CT method (2^−ΔΔCt^) (*P < 0.05). **(B)** The cell viability was measured by CCK-8 assays in the treated MCF-7 cells (*P < 0.05, vs. control group). **(C)** The cell viability was measured by CCK-8 assays in the treated MDA-MB-453 cells (*P < 0.05, vs. control group). **(D, E)** Colony forming assays were performed to detect cell proliferation (*P < 0.05, vs. NC group). **(F, G)** The cycle distribution was detected by flow cytometry, and cells in G0/G1, S and G2/M phases were quantified (*P < 0.05). **(H)** Cell apoptosis was detected using an Annexin V-FITC/7AAD double staining kit, and the percentages of positive apoptotic cells was determined by flow cytometry (*P < 0.05, vs. NC group).

**Figure 3:**
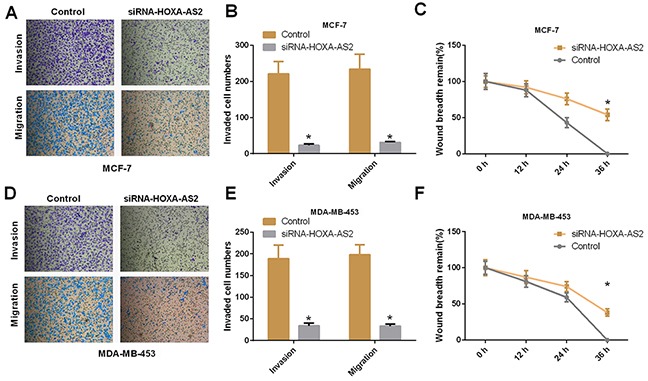
Silencing of HOXA-AS2 expression inhibits migration and invasion of breast cancer cells **(A, B)** Migratory and invasive capacities of MCF-7 cells were detected by transwell invasion and transwell migration assays (*P < 0.05). **(C)** Scratch-healing assays were used to assess MCF-7 cell migration. The remaining wound size was detected at 0, 12, 24, and 36 hrs (*P < 0.05). **(D-F)** Migratory and invasive capacities of MDA-MB-453 cells were determined by transwell invasion, migration and scratch-healing assays (*P < 0.05).

Then, we designed a siRNA to knock down HOXA-AS2 in MCF-7 and MDA-MB-453 cells. qRT-PCR was used to determine the efficiencies of HOXA-AS2 siRNAs (*P* < 0.05, Figure 2A). Using CCK-8 assays, we found that silencing of HOXA-AS2 inhibited MCF-7 and MDA-MB-453 cell proliferation (*P* < 0.05, Figure 2B, 2C). Furthermore, colony forming assays also indicated that silencing of HOXA-AS2 inhibited the proliferation of MCF-7 and MDA-MB-453 cells (*P* < 0.05, Figure 2D, 2E). We the analyzed cell cycle distribution using flow cytometry with propidium iodide staining of siRNA-HOXA-AS2-treated MCF-7 and MDA-MB-453 cells. Compared with control cells, knockdown of HOXA-AS2 caused cell cycle arrest in G0/G1 phase 48 hrs after transfection (*P* < 0.05, Figure 2F, 2G). We also investigated the effect of HOXA-AS2 siRNAs on cell apoptosis. The pro-apoptotic indexes of knockdown control cells and HOXA-AS2-silenced cells (siRNA-HOXA-AS2) were 2.97% and 20.% (MCF-7, *P* < 0.05), and 3.87% and 22.1% (MDA-MB-453, *P* < 0.05), respectively (Figure 2H). Moreover, the migratory and invasive capacities of MCF-7 cells decreased after HOXA-AS2 knockdown, as shown by transwell invasion, transwell migration, and scratch-healing assays (*P* < 0.05, Figure 3A-3C). Similarly, we detected the same trend in MDA-Mb-453 cells (*P* < 0.05, Figure 3D, 3E).

### Silencing of HOXA-AS2 expression suppresses tumor growth and inhibits Ki-67 expression *in vivo*

**Figure 4:**
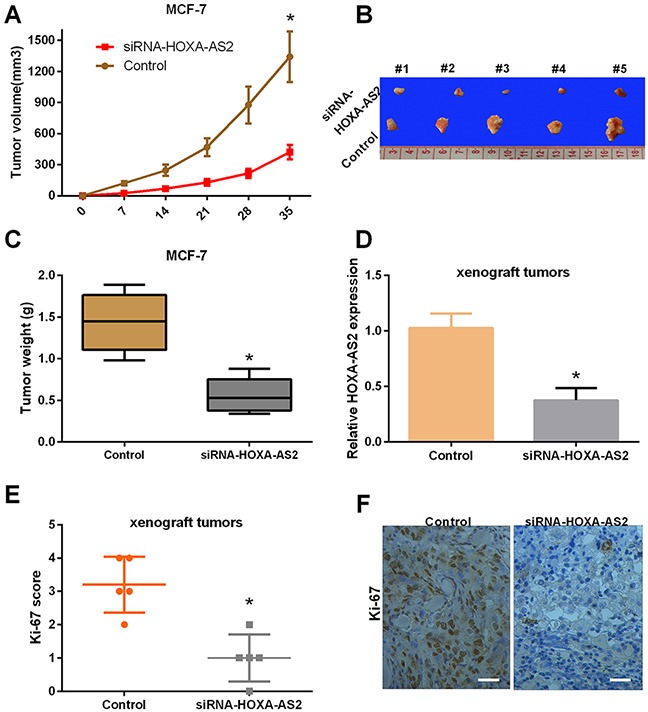
Silencing of HOXA-AS2 expression suppresses tumor growth and inhibits Ki-67 expression *in vivo* **(A)** Silencing of HOXA-AS2 expression reduced the tumor volume. Athymic mice were injected with MCF-7 cells transfected with control or HOXA-AS2 siRNAs. Tumor volume was measured at 0, 7, 14, 21, 28 and 35 days (*P < 0.05). **(B)** Photographs of representative tumors in nude mice were obtained five weeks after injection. **(C)** The tumor weight was calculated (*P < 0.05). **(D)** The expression level of HOXA-AS2 was detected by qRT-PCR in tissues (*P < 0.05). **(E, F)** The protein expression level of Ki-67 was analyzed by immunohistochemical assays (*P < 0.05).

To investigate the effects of siRNA-HOXA-AS2 on the growth of breast cancer *in vivo*, MCF-7 cells were transfected with control or HOXA-AS2 siRNAs and were subcutaneously injected into athymic mice. Our results showed that tumors from the siRNA-HOXA-AS2-transfected MCF-7 cells grew slower than that of the control group during the whole tumor growth period (*P* < 0.05, Figure 4A). Five weeks after inoculation, the tumors were excised, and photographs of the excised tumors were obtained (Figure 4B). The average weight of tumors derived from siRNA-HOXA-AS2-transfected MCF-7 cells was significantly smaller than those of control groups (*P* < 0.05, Figure 4C). Furthermore, the HOXA-AS2 and Ki-67 levels were detected by qRT-PCR and immunostaining. The results indicated that HOXA-AS2 expression in tumors of the siRNA-HOXA-AS2-treated group was significantly lower than the control group (*P* < 0.05, Figure 4D). Additionally, Ki-67 expression was significantly decreased in tumors of the siRNA-HOXA-AS2-treated group compared with the control group (*P* < 0.05, Figure 4E, 4F). Therefore, our results demonstrated that silencing of HOXA-AS2 expression can inhibit proliferation of breast cancer cells *in vivo*.

### MiR-520c-3p was regulated by HOXA-AS2 in breast cancer

**Figure 5:**
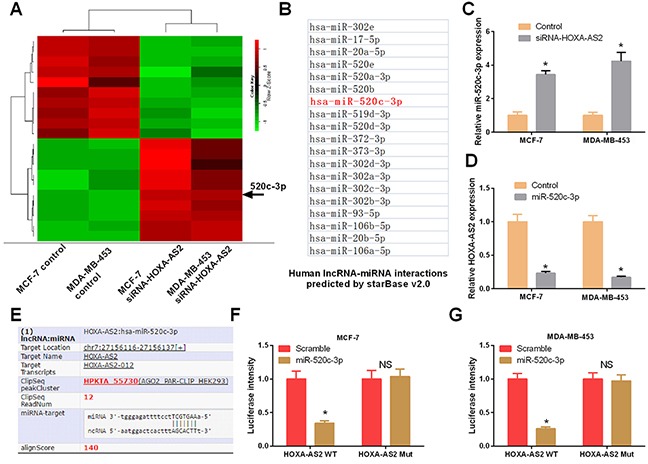
Mir-520c-3p was directly regulated by HOXA-AS2 in breast cancer **(A)** MCF-7 and MDA-MB-453 cells were transfected with HOXA-AS2 siRNAs or control for 48 hrs. Hierarchical clustering revealed systematic variations in the expression of miRNAs. Numerous differentially expressed miRNAs between control and HOXA-AS2 siRNAs transfected MCF-7 and MDA-MB-453 cells are shown on a scale from green (low) to red (high). The arrow indicates miR-520c-3p, which was included in these miRNAs. **(B)** StarBase v2.0 predicted the HOXA-AS2-regulated miRNAs (red one was miR-520c-3p). **(C)** qRT-PCR analysis of miR-520c-3p expression in MCF-7 and MDA-MB-453 cells transfected with HOXA-AS2 siRNAs or control (*P < 0.05). **(D)** qRT-PCR analysis of HOXA-AS2 expression in MCF-7 and MDA-MB-453 cells transfected with miR-520c-3p mimics or control (*P < 0.05). **(E)** StarBase v2.0 results showing the sequence of HOXA-AS2 with highly conserved putative miR-520c-3p binding sites. **(F, G)** MCF-7 and MDA-MB-453 cells were co-transfected with the reporter plasmid (or the corresponding mutant reporter) and miR-520c-3p. The relative fluorescence value was detected by luciferase reporter gene assays (*P < 0.05).

To investigate the effect of HOXA-AS2 on the expression of miRNAs, MCF-7 and MDA-MB-453 cells were transfected with HOXA-AS2 siRNAs and controls for 48 hrs. Hierarchical clustering of differentially expressed miRNAs is shown in Figure 5A. Then, starBase v2.0 software was used to predict the miRNAs that interacted with HOXA-AS2 (Figure 5B). MiR-520c-3p was identified in both array results and bioinformatics analysis results; thus, it was chosen for further study. To explore the regulatory relationship between HOXA-AS2 and miR-520c-3p, MCF-7 and MDA-MB-453 cells were transfected with HOXA-AS2 siRNAs and controls, and qRT-PCR was used to detect the expression level of miR-520c-3p. The results showed that silencing of HOXA-AS2 increased the expression level of miR-520c-3p in MCF-7 and MDA-MB-453 cells (*P* < 0.05, Figure 5C). Meanwhile, miR-520c-3p also inhibited the expression of HOXA-AS2 in MCF-7 and MDA-MB-453 cells (*P* < 0.05, Figure 5D). These results indicate that HOXA-AS2 and miR-520c-3p inhibit each other in breast cancer. As shown in Figure 5E, there was a putative miR-520c-3p target site in the HOXA-AS2 sequence. To confirm this interaction, the wild type sequence of HOXA-AS2 or its mutant sequence was subcloned into the pMIR luciferase reporter. MCF-7 and MDA-MB-453 cells were co-transfected with the reporter plasmid (or the corresponding mutant reporter) and miR-520c-3p. Our results indicated that the relative luciferase activity of the pMIR-HOXA-AS2-wt construct was significantly decreased but was abolished in HOXA-AS2-mt-transfected MCF-7 (*P* < 0.05, Figure 5F) and MDA-MB-453 cells (*P* < 0.05, Figure 5G). In addition, the relationships between miR-520c-3p expression and clinical characteristics of 38 patients with breast cancer were analyzed, and we found that miR-520c-3p was significantly associated with invasion (*P* = 0.018), lymphatic metastasis (*P* = 0.0003), distant metastasis (*P* = 0.0016), and TNM stage (*P* = 0.0003), which has a negative relationship with HOXA-AS2 (Table 2).

**Table 2:** Correlation analysis between miR-520c-3p expression and clinic pathological characteristics of patients with breast cancer

Characteristics	No. of patients	Mean ± SD	*P* value
**Total no. of patients**	38		
**Age (year)**			
>60	28 (73.7%)	11.87 ± 1.73	0.435
≤60	10 (26.3%)	12.36 ± 1.54	
**Invasion**			
T0-T2	21 (55.3%)	11.26 ± 1.57	0.018*
T3-T4	17 (44.7%)	12.45 ± 1.35	
**Lymphatic metastasis**			
N0	23 (60.5%)	10.29 ± 1.34	0.0003***
N1-N3	15 (39.5%)	12.16 ± 1.52	
**Distal metastasis**			
M0	27 (71.1%)	10.47 ± 1.42	0.0016**
M1	11 (28.9%)	12.18 ± 1.36	
**TNM stage**			
0 & I & II	23 (60.5%)	10.29 ± 1.34	0.0003***
III & IV	15 (39.5%)	12.16 ± 1.52	

*Indicated statistical significance (*P* < 0.05), **indicated statistical significance (*P* < 0.01), ***indicated statistical significance (*P* < 0.001).

### HOXA-AS2 controls the targets of miR-520c-3p in breast cancer

**Figure 6:**
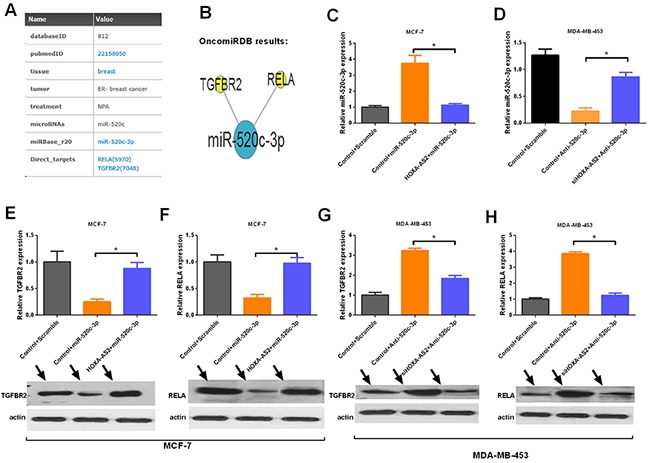
HOXA-AS2 controls the expression of the targets of miR-520c-3p **(A)** The OncomiRDB database supplied the direct targets (TGFBR2 and RELA) of miR-520c-3p. **(B)** The OncomiRDB regulatory network showed the regulatory relationships of miR-520c-3p, TGFBR2 and RELA. **(C, D)** qRT-PCR analysis of miR-520c-3p expression in MCF-7 and MDA-MB-453 cells transfected with control plus scramble, control plus miR-520c-3p, and HOXA-AS2 plus miR-520c-3p (*P < 0.05). **(E-H)** qRT-PCR (up panel) and Western blot (down panel) analysis of TGFBR2 and RELA expression in breast cancer cells treated with various vectors (*P < 0.05).

To further investigate the regulatory effect of HOXA-AS2 on miR-520c-3p, the OncomiRDB Database was used to search for direct targets of miR-520c-3p in breast cancer. As shown in Figure 6A, we identified TGFBR2 and RELA as direct targets of miR-520c-3p in breast cancer. The network from OncomiRDB shows the relationships among miR-520c-3p, TGFBR2 and RELA (Figure 6B). Then, we artificially altered the expression level of miR-520-3p by HOXA-AS2, si-HOXA-AS2, miR-520c-3p or antisense miR-520c-3p (Anti-520-3p) in breast cancer cells, and qRT-PCR was used to validate the transfection efficiency (*P* < 0.05, Figure 6C, 6D). We found that HOXA-AS2 controls the expression level of TGFBR2 and RELA in MCF-7 cells (*P* < 0.05, Figure 6E, 6F). Similar results were observed in MD-MB-453 cells (*P* < 0.05, Figure 6G, 6H).

### MiR-520c-3p rescued the si-HOXA-AS2-induced tumorigenesis inhibition of breast cancer

**Figure 7:**
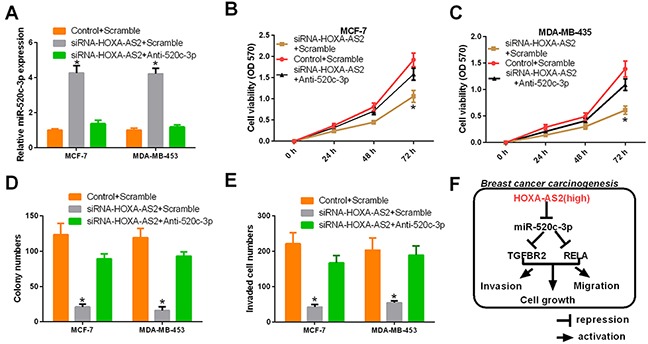
HOXA-AS2 promotes proliferation and invasion of breast cancer by miR-520c-3p MCF-7 and MDA-MB-453 cells were transfected with control plus scramble, HOXA-AS2 siRNAs plus scramble or HOXA-AS2 siRNAs plus anti-miR-520c-3p. **(A)** The expression level of HOXA-AS2 was detected by qRT-PCR and normalized to GAPDH in MCF-7 and MDA-MB-453 cells (*P < 0.05). **(B, C)** CCK-8 assays were used to measure the proliferation of treated MCF-7 and MDA-MB-453 cells (*P < 0.05). **(D)** Colony forming assays were performed to analyze the cell proliferation of treated MCF-7 and MDA-MB-453 cells (*P < 0.05). **(E)** Transwell assays were performed to detect the invasion of treated MCF-7 and MDA-MB-453 cells (*P < 0.05). **(F)** Schematic representation of the regulatory mechanism involved in the HOXA-AS2/miR-520c-3p axis in breast cancer.

Then, we performed a functional analysis of the HOXA-AS2/miR-520c-3p axis in breast cancer cell carcinogenesis. MCF-7 and MDA-MB-453 cells were transfected with control plus scramble sequences, HOXA-AS2 siRNAs plus scramble sequences or HOXA-AS2 siRNAs plus anti-miR-520c-3p. The results revealed that HOXA-AS2 siRNAs significantly increased the miR-520c-3p expression in breast cancer cells, which was rescued by anti-520c-3p (*P* < 0.05, Figure 7A). Moreover, CCK-8 results indicated that HOXA-AS2 silencing prevented the proliferation of MCF-7 cells, but this was rescued by anti-520c-3p (*P* < 0.05, Figure 7B). Similar findings were also observed in MDA-MB-453 cells (*P* < 0.05, Figure 7C). Colony forming assay results showed that HOXA-AS2 silencing inhibited cell proliferation and was rescued by anti-520c-3p (*P* < 0.05, Figure 7D). Furthermore, we found that HOXA-AS2 silencing inhibited cell invasion, but invasion was rescued by anti-520c-3p in MCF-7 and MDA-MB-453 cells (*P* < 0.05, Figure 7E). In conclusion, we found that highly expressed HOXA-AS2 down-regulates miR-520c-3p, then release its targets, TGFBR2 and RELA, and promotes proliferation, migration and invasion of breast cancer cells (Figure 7F).

## DISCUSSION

As the most common gynecological cancer, which has a low 5-year survival rate in patients with metastasis, breast cancer research is urgently needed to improve early diagnosis and treatment in human populations [35]. Non-coding RNA (ncRNA) has been regarded as a mechanism potentially driving breast cancer progression and metastasis and may serve as a new and more effective therapy in breast cancer [36, 37]. Among these ncRNAs, lncRNAs, which have extensive functions in cancer development [38, 39], have emerged as a new hot topic in early diagnosis and molecular targeted therapy [40]. At present, the relationship between lncRNA HOXA-AS2 and breast cancer is unclear. In this study, our results showed that HOXA-AS2 was up-regulated in breast cancer and associated with invasion, lymphatic metastasis, distant metastasis, TNM stage, and postoperative survival. Functional studies indicated a role of HOXA-AS2 in cell proliferation, apoptosis, migration and invasion of breast cancer cells. We also found that HOXA-AS2 silencing dramatically suppressed cell proliferation *in vivo*. These results indicated that HOXA-AS2 may be a good early diagnosis biomarker and therapeutic target in breast cancer. Ki-67, a marker of proliferation, is present in all proliferating tumor cells and is expressed in all stages of the cell cycle except G0 phase [41]. Ki-67 measurement is a standard method to evaluate the proliferative activity of tumor cells [42]. Several studies have shown that highly expressed Ki-67 is related to the development and clinicopathological features of breast cancer [43, 44]. In our study, we found that silencing of HOXA-AS2 inhibited Ki-67 protein expression in breast cancer cells, suggesting that HOXA-AS2 is closely involved in the proliferation of breast cancer and may be a critical marker along with Ki-67 in breast cancer diagnosis.

Previous studies have indicated that miRNAs have important biological functions in breast cancer, such as cell proliferation, apoptosis, differentiation, inflammation, and metastasis [45, 46]. In our study, we found that miR-520c-3p showed low expression and plays important roles in human breast cancer, which indicates it may be a potential biomarker in the diagnosis and treatment of breast cancer. Our findings enhanced the knowledge of miRNAs in this field. Studies have also indicated that lncRNAs potentially affect target gene levels as ceRNAs through miRNA response elements (MREs) [27]. At present, a number of lncRNAs that serve as ceRNAs have been shown to be involved in the occurrence and development of various diseases [47, 48]. In our study, we performed a microarray to screen the HOXA-AS2-regulated miRNAs in breast cancer. StarBase v2.0 was also used to predict the binding sites between HOXA-AS2 and the regulated miRNAs. Then, dual-luciferase reporter assays confirmed that miR-520c-3p, screened by microarray and bioinformatics analyses, was a HOXA-AS2-regulated miRNA in breast cancer cells. Functional studies demonstrated that HOXA-AS2 alters proliferation and invasion of breast cancer by regulating miR-520c-3p.

In the present study, we also explored the mechanism of the HOXA-AS2/miR-520c-3p axis in breast cancer and detected the influences of HOXA-AS2 on the targets (TGFBR2 and RELA) of miR-520c-3p. We found that HOXA-AS2 controls the expression of TGFBR2 and RELA by acting as a miR-520c-3p sponge. A previous study indicated that TGF-β has a dual effect (inhibiting early-stage tumor growth but promoting tumor metastasis) in cancer development [49]. TGF-β2, which belongs to the TGF-β family, contributes to the recruitment and activation of SMAD family members that regulate gene expression [50]. Our research showed that HOXA-AS2/miR-520c-3p may participate in breast cancer tumorigenesis through the TGF-β signaling pathway. Another target of miR-520c-3p, RELA (p65, NF-κB3), is a member of the transcription factor nuclear factor kappa B (NF-κB) family. RELA was shown to regulate the growth and malignancy of various tumors by regulating pro-inflammatory and pro-survival genes [51]. Our results demonstrated that the HOXA-AS2/miR-520c-3p axis controls the expression of RELA, suggesting a role for the HOXA-AS2/miR-520c-3p in breast cancer through the NF-κB signaling pathway.

In conclusion, our study found the following: a) HOXA-AS2 was up-regulated in breast tissues and cell lines. b) HOXA-AS2 expression was associated with invasion, lymphatic metastasis, distant metastasis, TNM stage, and postoperative survival of breast cancer. c) Silencing of HOXA-AS2 significantly suppressed carcinogenesis of breast cancer. d) HOXA-AS2 acts as a molecular sponge for miR-520c-3p and regulates its targets. The HOXA-AS2/miR-520c-3p regulatory network may shed light on tumorigenesis in breast cancer and may contribute to the development of novel diagnostic and treatment approaches for breast cancer.

## MATERIALS AND METHODS

### Clinical specimens

A total of 38 patients who were diagnosed with breast cancer and had undergone a mammectomy between 2009 and 2015 in the Affiliated Tumor Hospital of Harbin Medical University were enrolled in the study. No patients received radiotherapy or chemotherapy before surgery. The study was approved by the Ethics Committee of the Affiliated Tumor Hospital of Harbin Medical University. Written informed consent was obtained from all patients in this study. The histological diagnosis of breast cancer was based on the World Health Organization (WHO) criteria. All samples (cancer and paired noncancerous tissues) were stored in liquid nitrogen with RNALater (Qiagen) immediately after resection and saved until RNA extraction.

### Cell culture

A human mammary epithelial cell line (MCF-10A) was obtained from the American Type Culture Collection (ATCC, Manassas, VA), and three types of breast cancer cell lines (MDA-MB-231, MDA-MB-453, and MCF-7) were purchased from the Type Culture Collection of the Chinese Academy of Sciences (Shanghai, China). MCF-10A cells were maintained in mammary epithelial growth medium (MEGM, serum-free, Clonetics) including 100 ng/ml cholera toxin. MCF-7 cells were maintained in Eagle's minimum essential medium (MEM, GIBCO) including 10% FBS. MDA-MB-453 cells were cultured in Dulbecco's modified Eagle's medium (DMEM, Invitrogen) with 10% FBS. MCF-10A, MDA-MB-453 and MCF-7 cells were cultured as monolayers in a humidified atmosphere of 5% CO_2_. MDA-MB-231 cells were cultured in Leibovitz's L-15 medium (GIBCO) supplemented with fetal bovine serum (FBS) (Sigma Aldrich), in a CO_2_-free culture incubator.

### Transfection of breast cancer cells

MCF-7 and MDA-MB-453 cells were treated with HOXA-AS2 siRNAs at a final concentration of 50 nM, plasmid vectors, and 50 nM of miR-520c-3p or anti-miR-520c-3p oligonucleotides using Lipofectamine 3000 (Invitrogen, Carlsbad, CA, USA) according to the manufacturer's protocol. The siRNA duplexes targeting HOXA-AS2 were designed and synthesized by Qiagen (Hilden, Germany). The miR-520c-3p and anti-miR-520c-3p oligonucleotides were purchased from GenePharma (Shanghai, China). Plasmid vectors (HOXA-AS2 and empty vector) for transfection were extracted using a DNA Midiprep kit (Qiagen, Hilden, Germany). The full-length complementary DNA of HOXA-AS2 was synthesized by GenScript (GenScript.com) and cloned into the pcDNA3.1 (+) vector (Invitrogen) according to the manufacturer's instructions.

### Quantitative real-time reverse transcription PCR (qRT-PCR)

Total RNA was isolated from tissues and cells using TRIzol reagent (Invitrogen, CA, USA) according to the manufacturer's instructions. First-strand cDNAs were reverse transcribed from total RNA using the RevertAid First Strand cDNA Synthesis kit (Thermo Fisher). Quantitative PCR was performed using the SYBR-Green PCR Master Mix kit (TaKaRa) and an ABI 7500 Real time PCR system (Applied Biosystems). The primers of the control genes GAPDH and U6 (internal loading control) were designed and are shown (Table 3).

**Table 3:** The specific primer sequences for quantitative polymerase chain reaction

ID	Primer sequences (5′ to 3′)
GAPDH F	TGTTCGTCATGGGTGTGAAC
GAPDH R	ATGGCATGGACTGTGGTCAT
U6 F	CTCGCTTCGGCAGCACA
U6R	AACGCTTCACGAATTTGCGT
HOXA-AS2 F	CCCGTAGGAAGAACCGATGA
HOXA-AS2 R	TTTAGGCCTTCGCAGACAGC
TGFBR2 F	CCATTCTTCTCAAGTCCCAAAG
TGFBR2 R	ATTTTTCTCCCACAAGGCAGTA
RELA F	TCTGCTTCCAGGTGACAGTG
RELA R	ATCTTGAGCTCGGCAGTGTT
miR-520c-3p F	GCCGCCAAAGTGCTTCCTTTTAG
miR-520c-3p R	TCGCACTGGATACGACACCCTC

### Western blot assay

Western blot assays were performed to determine the protein levels of TGFBR2 and RELA. The treated MCF-7 and MDA-MB-453 cells were lysed by Radio Immunoprecipitation Assay (RIPA) buffer (Thermo Scientific, Rockford, IL, USA) containing a protease inhibitor cocktail (Roche Diagnostics). The concentrations were detected using a BCA Protein Assay kit (Thermo Fisher Scientific., Rockford, IL, USA). Proteins were separated by 10% SDS/PAGE and transferred onto a polyvinylidene fluoride membrane (PVDF, Millipore, Billerica, MA). The PVDF membranes were blocked and incubated with primary antibody overnight at 4°C. Then, the PVDF membranes were incubated with HRP-conjugated secondary antibody. The results were obtained using an ECL detection kit (Millipore Corp. Bedford, MA). Primary antibodies against TGFBR2 (1:500, Abcam, ab-78419, United States), RELA (1:500, PTGlab, 10745-1-AP, United States), and actin (1:2000, Cell Signaling Technology, CST, Boston, USA) were used.

### miRNA target prediction

The analysis of HOXA-AS2-predicted miR-520c-3p targets was performed using starBase v2.0 (http://starbase.sysu.edu.cn/). The OncomiRDB database was used to identify direct targets of miR-520c-3p (http://bioinfo.au.tsinghua.edu.cn/member/jgu/oncomirdb/).

### Luciferase assay

MCF-7 and MDA-MB-453 cells (5 × 10^4^ cells/well) were seeded in 24-well plates and co-transfected with miR-520c-3p and wild type or mutant HOXA-AS2, along with a Renilla plasmid (RL-SV40) using Lipofectamine 3000 (Invitrogen). The pRL-CMV Renilla vector served as an internal control. Cell extracts were harvested 48 hrs post-transfection, and a Dual-Luciferase Reporter Assay System (Promega) was used to measure the reporter gene activities according to the manufacturer's instructions. All experiments were performed in triplicate and repeated twice.

### Proliferation assay

Cell proliferation was measured by cholecystokinin octapeptide (CCK-8) assays. The treated MCF-7 and MDA-MB-453 cells (3 × 10^3^ cells/well) were seeded in a 96-well plates and incubated at 37°C for 24, 48, and 72 hrs. Then, 10 μL CCK-8 (Dojindo Laboratories, Kumamoto, Japan) was added to each well at the indicated time point. After 4 hrs, an Elx800 Reader (Bio-Tek Instruments Inc., Winooski, VT, USA) was used to record the absorbance value at 570 nm. For colony formation assays, the treated MCF-7 and MDA-MB-453 cells (200 cells/well) were seeded in six-well plates and incubated at 37°C in complete medium for 14 days (culture medium was changed every three days). Cells were fixed and stained with 0.1% crystal violet. The number of colonies was counted.

### Flow cytometric analysis

For cell cycle assays, the treated MCF-7 and MDA-MB-453 cells were obtained, washed with PBS three times, fixed with 80% ethanol, subsequently incubated with RNase A (0.25 mg/ml, Sigma) for 30 min at 37°C, and then incubated with 20 μg/ml propidium iodide (KeyGen, Nanjing, China) for 20 min at room temperature. The cell cycle was analyzed by flow cytometry using a FACSCalibur Flow Cytometer (BD Biosciences, San Jose, CA, USA). The experiments were performed in triplicate. For cell apoptosis assays, the extent of apoptosis was measured using the Annexin V/7-AAD Apoptosis Detection Kit (Southern Biotechnology, Birmingham, Al, USA) according to the manufacturer's instructions. Cells (1 × 10^6^ cells/mL) were digested, centrifuged, washed with cold PBS twice, and re-suspended in 100 μL of 1× binding buffer. Then, they were double stained with Annexin V and 7-AAD and incubated at room temperature in the dark for 15 mins. The stained cells were analyzed by flow cytometry (BD Biosciences) using a FACSCalibur Flow Cytometer (BD Biosciences, San Jose, CA, USA). The experiments were performed in triplicate.

### Wound-healing assay

The wound healing assay was used to study cell migration. The treated MCF-7 and MDA-MB-453 cells (1 × 10^6^ cells) were seeded in 6-well plates and cultured in complete medium for 12 hrs at 37°C in a 5% CO_2_ incubator. A pipette tip was used to make a straight scratch, and the images of the scratch were obtained as baseline. Cells were cultured at 37°C for 48 hrs. The suspended cells were gently washed off twice with PBS. Photographs were obtained every 12 hrs for the next 36 hrs.

### Transwell assay

The cell invasive and migratory abilities were evaluated using transwell assays. For migration assays, 200 mL of treated MCF-7 and MDA-MB-453 cells (5 ×10^5^) with serum-free medium was seeded in the top 24-well Transwell chamber (8-μm pore; Corning, Painted Post, NY, USA) of the inserts, and 600 μL of complete medium was added to the lower chamber. After 24 hrs of incubation, the migratory cells were fixed, stained in 0.1% crystal violet solution, and counted under a confocal microscope. For invasion assays, the invasive cells were detected by Transwell Matrigel (BD Biosciences, San Diego, CA, USA) invasion assays. Matrigel (BD Biosciences, San Diego, CA, USA) was pre-coated onto the upper chambers. Other experimental procedures were similar to the migration assay.

### Tumor formation in nude mice

This study was approved by the Institutional Committee for Animal Research and performed according to the national guidelines for the care and use of laboratory animals. MCF-7 cells were transfected with HOXA-AS2 siRNAs and controls, and cells (1×10^6^ cells/mouse, 0.2 ml) were subcutaneously injected into the armpit of 6-week-old BALB/c athymic nude mice. Tumor volume and weight were measured at 0, 7, 14, 21, 28, and 35 days. The mice were euthanized 5 weeks after injection, and photographs of excised tumors were obtained. The excised tumors were then used into further analyses.

### Immunohistochemistry

Immunohistochemistry was performed on paraformaldehyde-fixed paraffin sections. The TMA blocks were then cut into 4 μM sections for immunostaining. The sections were incubated with Ki-67 (#9027; Cell Signaling) antibody overnight at 4°C. Histostain-Plus 3rd Gen IHC Detection Kit (Invitrogen Co., San Diego, CA) was applied for 30 mins to visualize the positive signals.

### MiRNA microarrays

MCF-7 and MDA-MB-453 cells were transfected with HOXA-AS2 siRNAs and control for 48 hrs. An Agilent human miRNA microarray (V2) was used to determine the expression levels of miRNAs according to the manufacturer's instructions. First, total RNA was extracted using TRIzol (Invitrogen) and dephosphorylated with calf intestine alkaline phosphatase (Amersham). The dephosphorylated RNA was denatured by DMSO (Sigma). Second, the labeled miRNAs were re-suspended in hybridization buffer (Agilent) and hybridized with a microarray (Agilent Technologies). Finally, images were analyzed using Agilent Feature Extraction software (version A.9.5.3; Agilent Technologies).

### Statistical analysis

Differences between groups were analyzed by Student's *t*-test using SPSS 21.0 software (SPSS Inc., Chicago, IL, USA). Survival curves were evaluated by the Kaplan-Meier method and compared by the log-rank test. *P* < 0.05 was considered statistically significantly. Data are shown as the mean ± standard deviation (SD), and all experiments were performed in triplicate.
